# Preschool Children’s Behavioral Tendency toward Social Indirect Reciprocity

**DOI:** 10.1371/journal.pone.0070915

**Published:** 2013-08-07

**Authors:** Mayuko Kato-Shimizu, Kenji Onishi, Tadahiro Kanazawa, Toshihiko Hinobayashi

**Affiliations:** Graduate School of Human Sciences, Osaka University, Suita, Osaka, Japan; Universidad Carlos III de Madrid, Spain

## Abstract

Social indirect reciprocity seems to be crucial in enabling large-scale cooperative networks among genetically unrelated individuals in humans. However, there are relatively few studies on social indirect reciprocity in children compared to adults. Investigating whether young children have a behavioral tendency toward social indirect reciprocity will help us understand how and when the fundamental ability to form cooperative relationships among adults is acquired. Using naturalistic observation at a nursery school, this study examined whether 5- to 6-year-olds show a behavioral tendency to engage in social indirect reciprocity in response to their peers’ prosocial behavior toward a third party. The results revealed that bystander children tended to display prosocial behavior toward their peers more frequently after observing these peers’ prosocial behavior toward third-party peers, compared with control situations; this suggests that 5- to 6-year-olds may have an essential behavioral tendency to establish social indirect reciprocity when interacting with peers in their daily lives. In addition, bystanders tended to display affiliative behavior after observing focal children’s prosocial behavior. In other words, observing peers’ prosocial behavior toward third-party peers evoked bystanders’ positive emotions toward the helpers. Considering both the present results and previous findings, we speculate that in preschoolers, such positive emotions might mediate the increase in the bystander’s prosocial behavior toward the helper. In addition, an intuitional emotional process plays an important role in the preschooler’s behavioral tendency toward social indirect reciprocity in natural interactions with peers.

## Introduction

The tendency for genetically unrelated individuals to build large-scale cooperative networks in human societies is a major exception in the animal kingdom [1]. Researchers have suggested that the principle of indirect reciprocity–the idea that altruistic (or prosocial) behavior toward an individual is returned by another individual–is crucial in enabling these cooperative networks [2], [3]. Three different forms of indirect reciprocity exist: social indirect (downstream) [4]–[6], generalized (upstream) [7], [8], and generalized indirect [3]. In this study, we focus on social indirect reciprocity (SIR), which means that if A helps B, then C will help A, who acted cooperatively toward B; this is based on individuals’ evaluations of others’ prior behaviors toward third parties [4]–[6]. SIR is associated with social evaluation or moral judgment in humans and seems to be most important form for human prosociality. SIR is more elaborate than the other two forms of indirect reciprocity and requires individuals to recognize and select those with whom they cooperate [2], [3]. Through computer simulations and analytic models, previous studies have demonstrated that SIR could evolve when individuals act according to particular strategies [2], [6]. In all such strategies, individuals have the tendency (1) to reward helpful individuals and (2) to detect and avoid helping cheaters [2], [6].

In reality, studies with human adults have demonstrated a behavioral tendency toward SIR in the decision to cooperate or defect in game experiments [9], [10]. However, there are relatively few studies on SIR in children. Therefore, investigating whether young children have a tendency toward SIR, as well as the manner in which such reciprocity develops during the early developmental stages, will help us understand how and when this tendency, that is so fundamental in organizing cooperative interactions between adults, takes root in people’s lives.

Prosocial behavior can be observed from the first year of a child’s life [11] and becomes common between ages 1 and 2 [12]. Additionally, even 14-month-olds have been shown to be capable of helping others achieve their goals [13]. However, this early prosocial tendency does not seem to be selective with regard to recipients [14], [15]. Such selectivity begins to appear between toddlerhood and the preschool period. For example, prosocial behavior becomes selective in terms of partners’ gender and personality [11], [16], familiarity between partners [17], [18], or the existence of prior prosocial behavior from the partners, thereby suggesting that children engage in direct reciprocity [19]–[23]. However, this selectivity is based on the partners’ own characteristics or behavior toward the potential helper itself. In order to build cooperative relationships through SIR, children require a more elaborate selective ability based on the social evaluation of a partner’s behavior toward a third party.

Recently, some studies have reported that young children have a tendency toward SIR. Experimental research has shown that infants can distinguish between puppets based on their behavior toward other puppets from the age of 3 months [24] and prefer to reach for helping puppets rather than hindering puppets from the age of 6 months onward [25]. These studies suggest that infants have the ability to evaluate others according to their prosocial behavior toward a third party from an early developmental phase (but see [26] for an opposing view).

Some studies have investigated whether young children use this ability in the early developmental stages. Olson and Spelke [27] examined how children helped a protagonist doll allocate resources to other dolls; 3.5-year-olds allocated more of the protagonist doll’s resources to dolls who had shared resources with a different doll than to dolls who had kept the resources for themselves, suggesting that young children do show SIR, at least in a resource-distribution paradigm. Vaish, Carpenter, and Tomasello [28] found that 3-year-old children helped a neutral adult actor more often than an actor who intended but failed to harm a third party. The children also helped accidentally harmful and neutral actors equally often. These results suggest that children’s prosocial behavior is influenced by their evaluation of not only the outcomes, but also of the intentions behind others’ actions toward a third party. Furthermore, Kenward and Dahl [29] explored how children distribute resources between helpful and harmful puppets and whether these children could explain their distributive behavior; 4.5-year-olds tended to give more to the helpful puppets and could justify their unequal distributions by referring to the helpful puppet’s prosociality or the harmful puppet’s antisociality. This result suggests that children might reward or punish a puppet by evaluating its moral behavior toward a third party. From these studies, it is apparent that young children have high levels of social evaluative abilities and that they use these abilities when judging others as worthy recipients of prosocial behavior; this is essential to establishing SIR. However, these findings are limited to children’s interactions with puppets or adult actors in controlled situations and cannot be generalized to children’s interactions with their peers in real life.

Researchers have argued that children’s prosocial behavior differs depending on whether it is directed toward peers or adults [30]. They have also emphasized that it is among peers that children learn to execute the principles of social exchange [31], and that peer relations are characterized by interactions involving equality and prosociality [32], [33]. Therefore, for a clear and complete picture of children’s cooperative interactions through SIR, we need to examine children’s behavioral tendencies toward their own peers who have acted prosocially toward other peers. To the best of our knowledge, no study has examined whether children display such behavioral tendencies in real-life situations. Thus, in this study, we examined whether preschool children have a behavioral tendency to cooperate through SIR in the natural setting of a nursery school. Specifically, we sought to confirm the hypothesis that a bystander child will act more prosocially or affiliatively toward a peer after observing that peer’s prosocial behavior toward another peer than the same children would in a control situation.

The abilities of social evaluation or moral judgment seem to be important for establishing SIR. However, the psychological processes underlying social evaluation with regard to SIR in preschoolers have yet to be completely elucidated. By the age of 3–3.5 years, children can act according to their own social evaluations derived from third parties’ previously observed social actions [27], [28]. However, in the interview study, preschoolers were unlikely to be capable of explicit reasoning, suggesting that preschoolers cannot yet explicitly understand principles such as direct and indirect reciprocity [34]. Haidt [35], [36] proposed a social intuitionist model of moral judgment according to which moral decisions can be directly influenced by automatically generated intuitions and conscious explicit reasoning processes occur mainly after these decisions have already been made even in adults. Taking these studies into consideration, Kenward and Dahl [29] indicated that a psychological process of social evaluation in 4.5-year-old children seems to be emotional and intuitional (children simply “like” or “hate” or they may apply more a specific rule that “reward” or “punish,” but the latter is also judged through automatic processing), although they can justify their social indirect reciprocal distribution later on.

In this study, we considered whether emotional processes influence bystander children’s behavioral tendency toward SIR in the daily natural interactions of 5- to 6-year-olds. We recoded bystander children’s affiliative behavior as an index of positive emotions because young children generally have positive emotions toward partners at whom they are directing affiliative behavior [37]. If bystander children show affiliative behavior toward a peer who has acted in a prosocial manner toward other children, then the bystander children likely feel positive emotions toward the prosocial peer.

To strictly investigate the above predictions, we have to partial out the effects of potential factors which could affect subjects’ prosocial or affiliative behavior as possible. Therefore, we adopted the assessment of differing social outcomes in similar contexts, contrasting the occurrence and nonoccurrence of prosocial behavior by peers. Recently, the control procedure [38] has been applied in studies focusing on children [39]–[42]. This control procedure allows for a conclusive interpretation of the effect of peers’ prosocial behavior toward third parties on the subsequent responses of bystander children. In addition, this procedure allows for within-child comparisons. Various individual characteristics, such as sociability [43], assertiveness [44], affect the occurrence of prosocial behavior among peers. By using within-child comparisons, we can compare the same children’s interaction in two contexts, thereby eliminating the influences of individual differences.

Bystanders’ behavioral tendencies may nevertheless be affected by the following factors. The first factor is the degree of familiarity between children. Laursen and Hartup [45] emphasized the differences in prosocial interaction depending on the degree of familiarity; other studies have also established this [17], [18], [46]. The second factor is the amount of prosocial or affiliative behavior that the peers receive in their everyday lives. Observational studies in preschool classrooms have reported individual differences in receiving positive social behavior, including prosocial and affiliative behaviors [47], [48]. In order to control for the potential influences of these two factors, we assessed and analyzed them.

## Materials and Methods

### Participants

This study was conducted at a private nursery school in Osaka prefecture, Japan. There were two classes of 5- to 6-year-olds: Class A (19 boys and 19 girls, mean age = 67.5 months, SD = 3.6) and Class B (16 boys and 16 girls, mean age = 67.3 months, SD = 3.7). A previous study indicated that significant increases in prosocial behavior occur as children pass through preschool from 3 to 6 years of age [49]. We needed to observe a large number of prosocial behaviors in order to analyze them quantitatively; therefore, we decided to target 5- to 6-year-old children as our participants. Because some children showed no prosocial behavior, the focal subjects were selected according to the frequency with which they exhibited prosocial behavior in preliminary observations. For the preliminary observations, an observer conducted an event sampling of prosocial behavior in Class A for 10 days. Six boys and 6 girls belonging to Class A were selected to be our focal subjects (mean age = 69.2 months, SD = 3.2) from among the children who displayed a higher frequency of prosocial behavior. Throughout the entire observation, every child (N = 69) who belonged to Classes A and B, except for the focal child observed in each session, had the potential to become a bystander as long as their behavior was used to test our hypothesis. In addition, we dealt with the bias of the focal children as much as possible by using within-subjects comparison and the generalized linear mixed model (GLMM) method. Therefore, we do not believe there to be problems about the sample size of this study. All participants spoke Japanese as their first language.

Prior to beginning the observations, the parents of all the children who belonged to the two subject classes of 5- to 6-year-olds were informed about the study and asked for consent; we obtained written consent from all the parents for their children’s participation in the study. The research complied with protocols approved by the ethical committee in the fields of Psychology and Behavioral Sciences of the Graduate School of Human Sciences at Osaka University. This study adhered to the Code of Ethics and Conduct of The Japanese Psychological Association.

### Procedure

Naturalistic observations were conducted during free play time in a classroom or playground four times a week between June 2009 and March 2010. During the study period, four female nursery staff members were present during the classroom and playground observation sessions. During free play time, the children of Classes A and B played together and could move freely between the two classes. The first author (MK), who is Japanese and had previously established a good rapport with the children and staff members in the nursery school, conducted all the observations alone in order to avoid any disturbance effect that could be caused by multiple observers. All observations were recorded through a handheld video recorder (SONY, DCR-SR60).

First, the observer used a focal sampling method [50] with each focal child serving as the target of the observations, in random order, for 5-min durations. When a focal child displayed prosocial behavior toward another child (a first-recipient), the observer immediately carried out a 10-min focal observation of that focal child as a post-prosocial (PP) observation. If a prosocial behavior from a focal child to a bystander child was observed during the 10-min observation, then the observation was terminated and excluded from the analysis. We did this because, in such a situation, we could not determine whether the bystander’s subsequent prosocial behavior was a behavioral tendency toward SIR or a direct reciprocation of the focal child’s prosocial behavior. The duration of the PP observation was based on a previous study that examined immediate exchange in direct reciprocity [17]. We considered the following two types of behavior to be prosocial behavior: object offering and helping (see Table 1 for definitions). These behaviors were chosen because other prosocial behaviors do not occur frequently during the preschool period [46], [49]. In this study, other prosocial behaviors, such as those that appear to benefit the initiator as well as the recipient (e.g., behaviors occurring as part of a play ritual) were seldom observed; consequently, these were not coded. Furthermore, behavior that was forceful or aggressive toward the recipient or was not welcomed by the recipient was not coded as prosocial behavior.

**Table 1:** Definitions of coded prosocial behaviors and affiliative behaviors.

Prosocial behaviors.	
Object offering	Giving objects to another child spontaneously, except in cases where the object is taken back within a minute
Helping	Assisting another child to accomplish some goal spontaneously (e.g., wearing a smock, assisting a horizontal bar or pushing a bicycle)
**Affiliative behaviors**	
Hand-to-body	Touching another child’s body spontaneously
Body-to-body	Clinging to another child’s body spontaneously
Talking	Talking to another child spontaneously, except in cases where verbal aggression is displayed (e.g., insults, derogatory comments). An instance of talking ended when a child stopped talking for more than five seconds, and another instance began when the child started talking again.
Showing	Showing objects to another child spontaneously
Approaching	Approaching within one meter of another child spontaneously, except in cases where the approach was accidental

The observer coded the following items during PP observation: (1) the name of the focal child who initiated the prosocial behavior, the first recipient, and the bystanders who were within 1 m of the focal child and who had observed the focal child’s prosocial behavior, and (2) the location (classroom or playground).

The observer also conducted a 10-min baseline observation (matched control: MC) of the same focal child for comparison with the PP observation. The observer conducted MC observations only when the following conditions were met: (1) the observations were within 2 h (before or after) of the start time of the PP observation on a different day (e.g., if a PP observation was conducted at 11∶00 a.m. on one day, the 10-min MC observation could be made between 9∶00 a.m. and 1∶00 p.m. on another day); (2) one of the bystanders from the PP observation, chosen at random, was within 2 m of the focal child (to reduce the possibility of the children not interacting in the MC observation because of the distance between the focal child and the bystander); (3) the focal child did not display any prosocial behavior toward the first recipient or a bystander during the 10-min MC observation; and (4) the location (classroom or playground) was the same.

The MC observation procedure ensured that the control data involved a close distance between the focal child and the bystander and that it was conducted at almost the same time and location as the PP observation. However, in the MC observation, to ensure efficiency in data collection, we set a control distance between the focal child and a bystander child (within 2 m) that was larger than the distance adopted in the PP observation (within 1 m). The observer coded the distance in meters between the focal child and each bystander child at the beginning of the MC observation in order to analyze separately those sessions in which bystanders were within 1 m of the focal child, and compare them with the analysis of all sessions. This was intended to determine whether the control distance (within 2 m) was valid.

Each MC observation was conducted at least 2 days after and within 14 days of the PP observation to allow us to consider the influence of any prosocial behavior observed during the PP observation on behavior during the MC observation, as well as to factor in any change in the relationship between the focal child and the bystander during the time lag.

In both the PP and MC observation sessions, we coded three types of items. The first were the bystander’s prosocial behaviors toward the focal child. The second were the five types of affiliative behavior from the bystander toward the focal child: hand-to-body, body-to-body, talking, showing, and approaching. These behaviors were mostly based on Fujisawa, Kutsukake, and Hasegawa [20]’s coding, though we excluded “look” (see Table 1 for definitions). We did not code affiliative behavior when it occurred within a series of interactions that contained a bystander’s prosocial behavior toward a focal child; that is, we did not code the bystanders’ affiliative behavior that accompanied their prosocial behavior. Finally, we noted the teacher’s involvement with the focal child or bystanders, including praising the focal child who had behaved prosocially toward another peer or encouraging bystanders to reward those who had behaved prosocially.

Familiarity between focal children and bystanders was assessed via an ethological method. Immediately after each PP or MC focal observation session was completed, the observer conducted a scan sampling and recorded the names of those peers within 1 m of each focal child using the instantaneous scan sampling method (average number of scan samplings per focal child = 509.33; range: 401–578) [50]. A proximity score was then calculated for every possible combination in the following manner: (the proximity score of a pair) = (the number of sampling points at which the pair was observed in proximity)/(the total data points for the focal child). Positive associations between familiarity and physical proximity have been reported in children [51]. In this study, the proximity score of a pair was used to define the degree of familiarity of that pair. The validity of this familiarity assessment has been confirmed in previous studies which found that pairs’ proximity scores were consistent with teachers’ friendship nominations [40], [41].

We also assessed the focal child’s usual frequency of receiving positive social behavior from peers. In order to assess this frequency, the observer conducted another focal observation of the focal child, separate from the PP-MC focal observations, and recorded the prosocial and affiliative behaviors from peers during that period (total observation time per focal child = 3430 min). Then, the usual frequencies of receiving prosocial and affiliative behaviors were calculated for each of the focal children.

### Reliability

In order to assess coding reliability, the first author and a well-trained research assistant independently coded a randomly selected portion (about 10%) of the video data (i.e., 65×10 min). The following are Cohen’s Kappa values for each category: object offering,.80; helping,.82; hand-to-body,.81; body-to-body,.79; talking,.78; showing,.72; approaching,.78; and location, 1.0. Inter-observer reliability ranged from good to excellent.

### Analysis

We investigated the differences in the number of prosocial and affiliative behaviors from the bystander children toward the focal children between the PP and MC observations. In this analysis, a PP-MC pair formed one data point. We used a within PP-MC observation pair GLMM [52] for analysis. The GLMM can handle non-normal data and contains random terms in its linear predictor. Random terms are used to represent subject-specific random variation and account for repeated sampling within the same individuals [52], [53]. Regarding sample size in this study, although the number of bystanders was sufficient, there were only 12 focal children. Therefore, we dealt with the bias of focal children by using within-subjects comparison and the GLMM method. In our GLMM models, the identities of the focal child and the bystanders were inserted as nested random effects to avoid the problem of non-independence that might occur if different dyads were formed by the same individuals.

In our analysis, we applied the same models 3 times using all datasets and the filtered data sets (Analyses 1–3). First, we analyzed the following 2 models using all 283 PP-MC pairs between 144 dyads (Analysis 1). Fifty-six children were included as bystanders in the completed models. In Model 1, we included the number of prosocial behaviors that emerged from the bystander (who had been randomly chosen as the subject of distance control in the MC observations) toward the focal child (or binary data indicating whether the bystander showed prosocial behavior toward the focal child) as a dependent variable with a Poisson (or binomial) error structure and log (or logit) link function, and the context (PP or MC), familiarity between focal children and bystanders, and the focal children’s usual frequency of receiving prosocial behavior as independent variables. The identities of the focal child and bystander were inserted as random terms in the model in order to avoid non-independence issues with “PP-MC pairs observed in the same dyads” or “different dyads formed by the same individuals.”

In Model 2, we included the number of affiliative behaviors performed by the bystander toward the focal child as a dependent variable, and the context (PP or MC), familiarity between focal children and bystanders, and the focal children’s usual frequency of receiving affiliative behavior as independent variables. The other parameters were the same as those in Model 1. In the analysis of prosocial behavior (Model 1), in addition to the analysis using the number of prosocial behaviors, we conducted another, similar analysis using binomial data to confirm whether similar results were obtained in both analyses.

Second, we analyzed the same models (Model 1 and Model 2) using the dataset filtered according to the following criteria (Analysis 2). In Analysis 2, we controlled three possibilities: 1) the possibility of imitation, 2) the influence of control distance at the starting point of the MC observations, and 3) the effect of class.

It is possible that bystanders were simply imitating other bystanders’ or a first recipient’s prior prosocial behavior toward the focal child. Previous studies have reported that children tend to imitate peers after observing those peers’ prosocial behavior [54]–[56]; that is, prosocial peer models are likely to elicit prosocial behavior among children. For SIR, it is important that bystanders selectively choose a child whom they have observed behaving prosocially toward another child to be the recipient of their prosocial or affiliative behavior. Thus, if bystanders chose a recipient of their prosocial or affiliative behavior not selectively but randomly, by imitation, this would not be evidence of SIR based on social evaluation. In order to eliminate this possibility, we conducted an analysis that omitted the data of PP-MC session pairs in which a first recipient displayed prosocial behavior toward a focal child ahead of a bystander and in which there were multiple bystanders in the PP observations.

We also had to consider the differences in control distance between focal children and bystanders at the starting point of the PP and MC observations. We needed to determine whether there was any difference between the analysis of the sessions in which a bystander was within 1 m at the start of MC observation and the analysis of all the sessions. We conducted Analysis 2 using only the data from situations in which a bystander was within 1 m of a focal child at the start of the MC observation.

In this study, the focal children were selected from Class A while bystanders comprised children from both Class A and Class B. Therefore, we were concerned that class membership might affect the degree of prosocial behavior between the focal child and the bystander, as this might have an impact on the results. Furthermore, the frequency of interactions and familiarity of children within the same class and between classes might be different. Therefore, we conducted Analysis 2 excluding the data of sessions in which a focal child and a bystander belonged to different classes; thus, we analyzed only the data of children in Class A. In addition, for the usual frequency of receiving positive social behavior, we used only those behaviors that focal children had received from others in the same class in the analysis.

Only the data that fit these strict control criteria (84 PP-MC pairs between 60 dyads) were used in Analysis 2; 12 children were included as focal children and 30 children were included as bystanders.

Third, we recalculated Analyses 1 and 2 using only the data in which bystanders were already interacting with a focal child (bystanders were conducting affiliative behavior toward focal children other than “approaching”) or were participating the same kind of play with focal children in the same play group when the MC observation began (Analysis 3). Analysis 3 was conducted in order to eliminate the possibility that bystanders simply noticed the focal children more in the PP observations than in the MC observations because the focal children performed noticeable social actions only at the start of the PP observations; thus, the bystanders were more likely to direct their future actions toward them in the PP observations. In the MC situations in Analysis 3, bystanders surely noticed the focal children and were equally likely to carry out prosocial or affiliative behavior as in the PP observations. One hundred ninety-one PP-MC pairs between 94 dyads (12 focal children and 44 bystanders) matched the criteria for the recalculation of Analysis 1, and 69 PP-MC pairs between 51 dyads (12 focal children and 26 bystanders) for the recalculation of Analysis 2.

The trend in the results was similar between Analyses 1–3, therefore we describe all three analyses in the Results section but, for the sake of brevity, only present specific values for Analysis 1 in the body text. In Model 1 (in Analyses1–3), no difference was found between using the number of prosocial behaviors or binary data for the dependent variable. Therefore, we present only the results based on the number of prosocial behaviors in this paper. All analyses were performed using SPSS20.0 statistical software.

## Results

### Descriptive Data

A total of 283 PP-MC pairs were recorded (mean: 23.58 PP-MC pairs per focal child, range: 20–26 PP-MC pairs).

The teacher’s involvement was checked for its potential influence on bystanders’ behavioral tendencies, in order to determine whether it should be controlled in subsequent analyses. Throughout the observations, no teacher’s involvement was recorded, suggesting that teachers did not frequently praise the prosocial children or encourage children to behave prosocially during free play time. Thus, it was not necessary to control for the influence of the teacher’s involvement.

### Analysis 1 (Using all Data): Model 1

**Figure 1:**
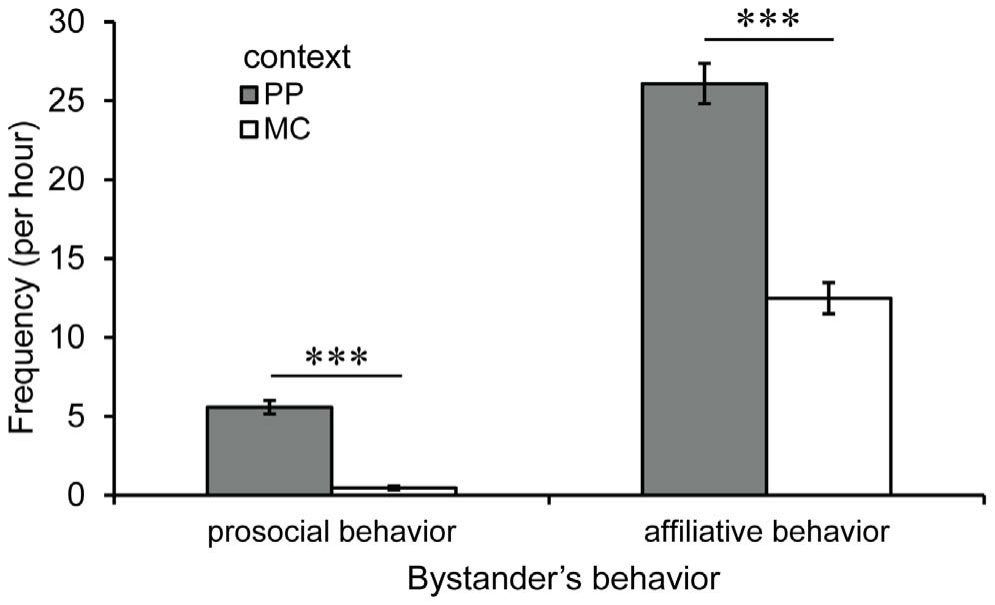
Actual frequencies of prosocial and affiliative behavior per hour in terms of context. Data are represented as session means ±1 SE of the actual measured value of frequencies of prosocial behavior and affiliative behavior for each context. ^***^ *p*<.001. The *p* values were calculated from the estimated values in Analysis1: Model 1 and Model 2.

The analysis in which Model 1 was applied revealed that the number of prosocial behaviors was significantly influenced by context, even when familiarity between focal children and bystanders and the focal children’s usual frequency of receiving prosocial behavior were simultaneously included as independent variables (Table 2). As shown in Figure 1, bystanders showed prosocial behaviors toward a focal child more frequently in PP observations than in MC observations, even though we controlled for familiarity and the usual frequency of receiving prosocial behavior.

**Table 2:** Influence of independent factors on the number of prosocial behavior from bystanders in Analysis 1, Model 1.

Independent term					
Factors	Level	Coef	SE (coef)	*t*	*P* (>|*t*|)
Intercept		−0.28	0.35	−0.80	0.42
Context	PP	2.48	0.24	10.24	<0.001
Familiarity between focal children and bystanders		1.51	0.93	1.61	0.11
The focal children’s usual frequency of receiving prosocial behavior		0.01	0.05	0.15	0.88

We analyzed the data in 566 sessions (283 PP-MC pairs, focal child = 12, bystander = 56, focal-bystander dyad = 144) in Analysis 1, Model 1. The generalized linear mixed model with Poisson error structures was used in the analysis. In the linear model with categorical independent variables, one of the levels was treated as a criterion, and the parameters of the other levels were estimated as the difference from the criterion level. In this model, in the factor “context”, the level “MC” was treated as a criterion, and the coefficient of “PP” was shown as the differences from the level of “MC”.

Familiarity between the focal children and bystanders and the focal children’s usual frequency of receiving prosocial behavior did not affect the total number of prosocial behaviors that focal children received from bystanders in the situations we observed.

### Analysis 1 (Using all Data): Model 2

The analysis in which Model 2 was applied revealed that the number of affiliative behaviors was significantly influenced by context, even when familiarity between the focal children and bystanders and the focal children’s usual frequency of receiving affiliative behavior were simultaneously included as independent variables (Table 3).

**Table 3:** Influence of independent factors on the number of affiliative behavior from bystanders in Analysis 1, Model 2.

Independent term					
Factors	Level	Coef	SE (coef)	*t*	*P* (>|*t*|)
Intercept		0.29	0.42	0.70	0.48
Context	PP	0.74	0.08	8.94	<0.001
Familiarity between focal children and bystanders		3.32	0.60	5.56	<0.001
The focal children’s usual frequency of receiving affiliative behavior		0.01	0.01	1.95	0.05

We analyzed the data in 566 sessions (283 PP-MC pairs, focal child = 12, bystander = 56, focal-bystander dyad = 144) in Analysis 1, Model 2. In the factor “context”, the parameters were shown in the same way as Table 2.

The focal child-bystander pairs with higher levels of familiarity showed more affiliative behaviors (received by focal children from bystanders). The focal children’s usual frequency of receiving affiliative behavior did not affect the total number of affiliative behaviors that focal children received from bystanders in the situations we observed.

As with our analysis of Model 1, bystanders showed affiliative behavior significantly more frequently toward a focal child in PP observations than in MC observations, even when we controlled for familiarity and the usual frequency of receiving affiliative behavior (Figure 1).

### Analysis 2 (Using the Filtered Data)

We also applied the same models in our analysis using the filtered data in order to eliminate the possibility of imitation, the influence of control distance at the start of the MC observations, and the effect of class. In this analysis, we examined 84 PP-MC pairs (mean: 7.00 PP-MC pairs per child, range: 4–11 pairs). The same trends as in Analysis 1 were confirmed, except for the influence of familiarity on analyzing the number of prosocial behaviors in Model 1 (Table S1), and the influence of the usual frequency of receiving affiliative behavior on analyzing the number of affiliative behaviors in Model 2 (Table S2). This analysis suggests that bystanders tended to engage in prosocial and affiliative behaviors toward peers more frequently soon after observing the peers’ prosocial behavior toward other peers than in control situations, even when the possibility that bystanders had imitated the first recipient’s or other bystanders’ prosocial behavior was eliminated.

### Analysis 3 (Recalculation of Analyses 1 and 2 using the Data in which by Standers Noticed Focal Children in the MC Observations)

We recalculated Analyses 1 and 2 using the data in which bystanders noticed focal children at the starting point of the MC observations (Analysis 3) in order to eliminate the possibility that the results of Analyses 1 and 2 were obtained because bystanders simply noticed the focal children more in the PP observations than in the MC observations. In this reanalysis, the same trends were confirmed as in Analyses 1 and 2 in the comparison between PP and MC in all models (Table S3–S6). This suggests that the bystanders had a behavioral tendency toward SIR even when we controlled for their awareness of the focal children.

## Discussion

In this study, we investigated whether preschool children, in their natural interactions, have a tendency to behave prosocially or affiliatively toward a peer after they have directly observed the peer behaving prosocially toward a third party.

The results showed that bystanders performed prosocial and affiliative behaviors toward focal children more frequently after the focal children’s prosocial behavior toward third parties, compared with control situations. This indicates that, 5- to 6-year-olds have a behavioral tendency toward prosocial and affiliative behaviors according to the recipient peers’ prior prosocial behavior toward another peer. Furthermore, the teachers neither praised the focal child who behaved prosocially toward peers nor encouraged bystander children to reward the focal child, suggesting that the children spontaneously displayed such a behavioral tendency.

At this age, the occurrence of prosocial behavior can be affected by various factors such as familiarity between children [17], [18] or their personalities [11], [16], [43], [44]. Therefore, in addition to the context (PP vs. MC), we included two factors as independent variables (familiarity between the focal children and bystanders and the focal children’s usual frequency of receiving positive social behavior), that seemed to affect the occurrence of prosocial behavior. As a result, the main effect of context was significant even if we included these additional factors in the models and partialed out their effects. Moreover, by using control procedures for observation and mixed model approaches (allowing for within PP-MC pair comparison and controlling the identities of the children) we avoided any possible effect of the various individual characteristics of each focal child or bystander on the behavioral tendency toward SIR. Thus, we exercised care in excluding the effects of children’s personalities and individual differences and the relationships between children on the behavioral tendency toward SIR.

The results have high ecological validity because we conducted naturalistic observations in a nursery school. However, it is difficult to collect abundant data in a naturalistic observational study. Therefore, to ensure efficiency in data collection, we established different control distances at the starting points of PP and MC observations and selected focal children from only one class. We then confirmed whether these variables had affected our results. The results of the analysis using the filtered data (Analysis 2) did not differ from the results when we analyzed all sessions (Analysis 1), indicating that neither control distance nor class affected our conclusion.

Moreover, we recalculated Analyses 1 and 2 using only the data in which bystanders noticed the focal children when the MC observation started (Analysis 3). The results of this analysis showed the same trends as in Analyses 1 and 2. This suggests that bystanders’ behavioral tendency toward SIR did not arise because bystanders simply noticed the focal children more in the PP observations than in the MC observations.

Based on these results, we conclude that preschool children have an essential behavioral tendency to establish SIR when interacting with their peers in naturalistic settings, as well as in their interactions with puppets or adult actors, as found in previous studies [27]–[29]. Our results, which extend the findings of previous studies [27]–[29], suggest that preschool children not only have the ability to evaluate partners on the basis of their prosocial behavior toward a third party, they can also use this ability in natural interactions with their peers. This study is the first to provide evidence indicating that children’s prosocial interactions can be formed for the benefits derived from exchanging prosocial behaviors according to the mechanism of SIR in natural interactions with their peers.

In this study, we also considered the psychological processes at work when children evaluate others’ behavior. Haidt [35], [36] proposed the social intuitionist model of moral judgment, in which moral decisions first arise as intuitions, and explicit reasoning occurs mainly after these decisions in order to justify them. In this model, the intuitional emotional process and the explicit reasoning process are not mutually exclusive and both processes function. First, the intuitional emotional process works to form simple evaluations (like or dislike, good or bad), then explicit reasoning process begins; this process can correct and override social evaluations from the intuitional emotional process. Some studies have supported this model. Using a neuroimaging method, a previous study showed that adults have nearly instant and automatic reactions to moral violations [57], suggesting the existence of intuitional processes for moral judgment. Unfair offers in the ultimatum game elicited brain activity in both areas related to emotion and cognition [58] and manipulating emotional state using hypnosis can change moral judgment [59]; these findings suggest that emotion plays a crucial role in moral judgment. Another study reported that when people solved difficult moral dilemmas that required a violation of their personal morals to achieve a greater good, they took more time to answer and showed activity in a brain region associated with internal conflict [60]. Moreover, some people who made a utilitarian moral judgment with a personal moral violation exhibited increased activity in the area of the brain associated with “cognitive” processes such as abstract reasoning and problem solving [60]. These results indicate that a conflict sometimes occurs between emotional and explicit reasoning processes, and that the latter can override initial intuitive emotional responses.

Previous studies suggest that preschoolers do not understand principles such as indirect reciprocity and fairness, but then children do begin to recognize these principles during the early elementary school years [34]. Kenward and Dahl [29] proposed two potential explanations for preschoolers’ moral judgment based on this viewpoint: (1) Children come to like the helper by observing their prosociality toward a third party, and then act prosocially toward the individual they came to like, or (2) They apply more specific rules than liking, such as “the helper should be rewarded” or “the non-helper should be punished,” but this evaluation automatically arises without an explicit moral reasoning process. Although our methodology did not allow us to interview children about their emotional states or explicit reasoning behind their behavior during the prosocial interactions, we found that bystanders tended to display not only prosocial behavior but also more affiliative behavior after observing focal children’s prosocial behavior. In this study, we did not code the bystanders’ affiliative behaviors that accompanied their prosocial behavior toward the focal children. Although the increase of affiliative behavior in the PP context was almost independent of that of the prosocial behavior, the mean number of affiliative behaviors per 10 min was 4.35 in the PP observations and 2.08 in the MC observations; two times higher in PP than in MC. Moreover, in Analysis 3, though many of the MC observations (137/191 sessions) had at least one affiliative behavior due to the filtering rule of data, the number of affiliative behaviors in the PP observations was still higher than in the MC observations. Considering both the results of this study and the previous findings, observing peers’ prosocial behavior toward third party peers seems to evoke bystanders’ positive emotions toward the helpers (e.g., bystanders come to like these helpers), and such positive emotions might mediate the increase in a bystander’s prosocial behavior toward a helper.

Comparative perspectives from other species, nonhuman primates in particular, help to understand evolution and the proximate psychological process of human prosociality. In a previous study, only after training, chimpanzees (*Pan troglodytes*) came to beg for food more often from an unfamiliar person who gave food to another (generous donor) than from one who refused to do so (selfish donor) [61]. Another study reported that chimpanzees spent more time in front of a human who gave food to another human than one who refused to do so [62]. However, the former could not eliminate the possibility that chimpanzees learned the relationship between the actors’ behavior pattern and getting more food [61], and the latter suggested that chimpanzees were evaluating the foraging opportunity [62]. Recently, using more appropriate experimental design, two studies clearly demonstrated that tufted capuchin monkeys (*Cebus apella*) accepted food less frequently from human actors who did not reciprocate in a social exchange with others [63] or from those who did not help another person [64]. In these studies, the monkeys observed social interactions between human actors and then chose one of the two actors to ask for food. The two actors always offered the identical food to the monkeys; therefore, the results of these studies were independent of food opportunity. This suggests that at least one nonhuman primate (tufted capuchin monkey) have the prerequisites for social evaluation to judge reciprocity or prosociality by observing social interactions between third parties.

Based on the studies of direct reciprocity and biological market theory in primates, direct reciprocity could be maintained by “emotionally based bookkeeping,” a system in which, when primates received services from other individuals, the experience triggered emotional evaluation of the individuals [65]. This emotional bookkeeping allows primates to maintain long-term tracking of reciprocal exchanges without an excessive cognitive load caused by storing all the specific exchanges in memory. This system may also work in social evaluations of social interactions between third parties in tufted capuchin monkeys.

The fact that capuchin monkeys possess the ability of social evaluation of social exchanges between third parties [63], [64] suggests that the explicit reasoning process (e.g. the understanding and use of the principle of fairness or indirect reciprocity) was not indispensable for such social evaluation associated with SIR. The social evaluation ability in humans may have developed from a lower-level process (emotional and intuition process) to a higher-level process (explicit moral reasoning process) in the course of human evolution, and now both of these processes are used in establishing SIR in human.

A change from the use of only the intuitional emotional process to the combination use of the intuitional emotional and explicit moral reasoning processes may work in human ontogeny similar to the human evolution process. Based on previous findings and those of the current study, we proposed a potential explanation for the preschooler’s psychological processes for SIR. Namely, at the age of 5 to 6, an intuitional emotional process plays an important role in children’s behavioral tendencies toward SIR when they are engaged in natural interaction with peers. Although, considering the results of previous studies, it seems relatively unlikely that an alternative explanation exists, perhaps preschool children use explicit moral reasoning like “the person who did the good deed should be rewarded depending on what they did” and they think “being friendly to the prosocial child” falls within the range of “rewarded” unlike the definition of altruistic behavior in adults. We cannot provide any data contributing to the body of knowledge on the topic of 5- to 6-years olds’ ability to use explicit reasoning in their behavioral tendency toward SIR. Therefore, future studies should investigate whether 5- to 6-year-olds can report their understanding of moral principles such as indirect reciprocity when they are asked to justify their behavioral tendency toward SIR. Moreover, the question of whether this explicit reasoning ability influences individual differences in the tendency toward SIR should be explored. Furthermore, measuring developmental changes in the neural activities of brain circuitry during moral judgment in preschoolers is the most effective approach.

Previous studies have suggested that young children tend to imitate their peers after observing these peers’ prosocial behavior [54]–[56]. Therefore, we conducted Analysis 2 to eliminate the possibility of a bystander child randomly selecting a recipient of prosocial behavior by simply imitating a first recipient’s or other bystander’s prior prosocial behavior toward the focal child. The results of Analysis 2 were similar to those of Analysis 1; that is, bystanders’ behavioral tendency toward SIR was not caused by their imitation of the first recipient or other bystanders.

However, we could not eliminate the possibility that the bystander imitated the focal child’s prosocial behavior toward the first recipient. If the bystander’s prosocial behavior was an imitation of the focal child randomly directed toward a nearby child, and if the behavioral tendency in this study is merely a byproduct of modeling, our conclusion would differ. However, if bystanders selected a focal child who behaved prosocially toward a third party as the recipient of their prosocial behavior, then our conclusion–that social evaluation affects bystanders’ behavioral tendencies–would not change. Our conclusion is supported by a previous study that used a resource distribution experiment to show that 4.5-year-olds had behavioral tendencies toward SIR; in addition, through a structured interview, the researchers found that these children socially evaluated a partner on the basis of that partner’s behavior toward third parties [29]. The present study further showed that a bystander’s affiliative behavior toward a focal child also tended to occur immediately after the focal child’s prosocial behavior, as compared with their behavior in control situations. This result supports the possibility that bystanders made their evaluations of prosocial partners on the basis of their positive emotions, which then influenced their behavioral tendencies.

In future studies, however, it will be necessary to eliminate any possibility that the bystanders’ behavioral tendency is a byproduct of modeling the focal children. To show this, we need to conduct focal observations of or interviews with bystanders. Such data could reveal whether bystanders’ prosocial behaviors are selectively directed to the child who behaves prosocially toward another peer.

SIR arises from two aspects of motivation: reward helpful individuals and avoid helping (or punish) harmful individuals [2], [4]–[6]. The present study explored only the behavioral tendency related to the former aspect and did not examine the latter. Previous studies have demonstrated that a “negativity bias” (a greater impact of negative information as compared with positive information) affects the behavioral tendencies of infants [24], [28], [66]. Vaish *et al*. [28] has shown that 3-year-olds’ prosocial behavior decreased toward a harmful individual but did not increase toward a helpful individual. Negativity bias has also been demonstrated in nonhuman primate: capuchin monkeys avoided non-reciprocal and non-helpful individuals with intentionality rather than express a preference for reciprocal or helpful ones [63], [64]. Compared with the behavioral tendency highlighted by the present study, the tendency to avoid helping (or to punish) a peer who behaves antisocially toward other peers may be seen more strongly and much earlier. In order to confirm this, 5- to 6-year-old children’s use of negative information to shape their behavior toward unhelpful peers should be investigated.

## Supporting Information

Table S1 **Influence of independent factors on the number of prosocial behavior from bystanders in Analysis 2, Model 1.**
(PDF)

Table S2 **Influence of independent factors on the number of affiliative behavior from bystanders in Analysis 2, Model 2.**
(PDF)

Table S3 **Influence of independent factors on the number of prosocial behavior from bystanders in Analysis 3 (recalculation of Analysis 1, Model 1).**
(PDF)

Table S4 **Influence of independent factors on the number of affiliative behavior from bystanders in Analysis 3 (recalculation of Analysis 1, Model 2).**
(PDF)

Table S5 **Influence of independent factors on the number of prosocial behavior from bystanders in Analysis 3 (recalculation of Analysis 2, Model 1).**
(PDF)

Table S6 **Influence of independent factors on the number of affiliative behavior from bystanders in Analysis 3 (recalculation of Analysis 2, Model 2).**
(PDF)
