# Supplementary material for: Preschool Children’s Behavioral Tendency toward Social Indirect Reciprocity
Source: PLoS One. 2013 Aug 7;8(8):e70915. doi: 10.1371/journal.pone.0070915 (PMC3737253; doi:10.1371/journal.pone.0070915)
Supplement: Table S1 — Influence of independent factors on the number of prosocial behavior from bystanders in Analysis 2, Model 1. (PDF) [file pone.0070915.s001.pdf]

Table S1: Influence of independent factors on the number of prosocial behavior from bystanders in Analysis 2, Model 1

Independent term Factors	Level	Coef	SE (coef)	t	P (>|t|)
Intercept		- 0.77	0.37	- 2.10	0.04
Context	PP	2.44	0.37	6.64	< 0.001
Familiarity between focal children and bystanders		4.28	1.67	2.56	0.01
The focal children's usual frequency of receiving prosocial behavior		< 0.01	0.12	- 0.01	0.99

We analyzed the data in 168 sessions (84 PP-MC pairs, focal child = 12, bystander = 30, focal-bystander dyad = 60) in Analysis 2, Model 1. In the factor "context", the parameters were shown in the same way as Table 2.
