# Supplementary material for: Preschool Children’s Behavioral Tendency toward Social Indirect Reciprocity
Source: PLoS One. 2013 Aug 7;8(8):e70915. doi: 10.1371/journal.pone.0070915 (PMC3737253; doi:10.1371/journal.pone.0070915)
Supplement: Table S2 — Influence of independent factors on the number of affiliative behavior from bystanders in Analysis 2, Model 2. (PDF) [file pone.0070915.s002.pdf]

Table S2: Influence of independent factors on the number of affiliative behavior from bystanders in Analysis 2, Model 2

Independent term					
Factors	Level	Coef	SE (coef)	*t*	*P* (>|*t*|)
Intercept		- 0.19	0.36	- 0.53	0.60
Context	PP	0.62	0.13	4.65	< 0.001
Familiarity between focal children and bystanders		1.78	0.81	2.21	0.03
The focal children's usual frequency of receiving affiliative behavior		0.02	0.01	4.26	< 0.001

We analyzed the data in 168 sessions (84 PP-MC pairs, focal child = 12, bystander = 30, focal-bystander dyad = 60) in Analysis 2, Model 2. In the factor "context", the parameters were shown in the same way as Table 2.
