# Supplementary material for: Preschool Children’s Behavioral Tendency toward Social Indirect Reciprocity
Source: PLoS One. 2013 Aug 7;8(8):e70915. doi: 10.1371/journal.pone.0070915 (PMC3737253; doi:10.1371/journal.pone.0070915)
Supplement: Table S3 — Influence of independent factors on the number of prosocial behavior from bystanders in Analysis 3 (recalculation of Analysis 1, Model 1). (PDF) [file pone.0070915.s003.pdf]

Table S3: Influence of independent factors on the number of prosocial behavior from bystanders in Analysis 3 (recalculation of Analysis 1, Model 1)

Independent term					
Factors	Level	Coef	SE (coef)	*t*	*P* (>|*t*|)
Intercept		- 0.35	0.41	- 0.85	0.39
Context	PP	2.10	0.26	8.03	< 0.001
Familiarity between focal children and bystanders		2.55	1.09	2.35	0.02
The focal children's usual frequency of receiving prosocial behavior		- 0.02	0.05	- 0.28	0.78

We analyzed the data in 382 sessions (191 PP-MC pairs, focal child = 12, bystander = 44, focal-bystander dyad = 94) in Analysis 3 (recalculation of Analysis 1, Model 1). In the factor "context", the parameters were shown in the same way as Table 2.
