# Supplementary material for: Preschool Children’s Behavioral Tendency toward Social Indirect Reciprocity
Source: PLoS One. 2013 Aug 7;8(8):e70915. doi: 10.1371/journal.pone.0070915 (PMC3737253; doi:10.1371/journal.pone.0070915)
Supplement: Table S4 — Influence of independent factors on the number of affiliative behavior from bystanders in Analysis 3 (recalculation of Analysis 1, Model 2). (PDF) [file pone.0070915.s004.pdf]

Table S4: Influence of independent factors on the number of affiliative behavior from bystanders in Analysis 3 (recalculation of Analysis 1, Model 2)

Independent term					
Factors	Level	Coef	SE (coef)	*t*	*P* (>|*t*|)
Intercept		0.15	0.48	0.32	0.75
Context	PP	0.45	0.09	5.22	< 0.001
Familiarity between focal children and bystanders		2.34	0.61	3.83	< 0.001
The focal children's usual frequency of receiving affiliative behavior		0.01	0.01	2.17	0.03

We analyzed the data in 382 sessions (191 PP-MC pairs, focal child = 12, bystander = 44, focal-bystander dyad = 94) in Analysis 3 (recalculation of Analysis 1, Model 2). In the factor "context", the parameters were shown in the same way as Table 2.
