# Supplementary material for: Preschool Children’s Behavioral Tendency toward Social Indirect Reciprocity
Source: PLoS One. 2013 Aug 7;8(8):e70915. doi: 10.1371/journal.pone.0070915 (PMC3737253; doi:10.1371/journal.pone.0070915)
Supplement: Table S5 — Influence of independent factors on the number of prosocial behavior from bystanders in Analysis 3 (recalculation of Analysis 2, Model 1). (PDF) [file pone.0070915.s005.pdf]

Table S5: Influence of independent factors on the number of prosocial behavior from bystanders in Analysis 3 (recalculation of Analysis 2, Model 1)

Independent term					
Factors	Level	Coef	SE (coef)	*t*	*P* (>|*t*|)
Intercept		- 0.11	0.67	- 0.17	0.87
Context	PP	2.27	0.38	5.98	< 0.001
Familiarity between focal children and bystanders		4.45	1.79	2.49	0.01
The focal children's usual frequency of receiving prosocial behavior		0.11	0.09	- 1.20	0.23

We analyzed the data in 138 sessions (69 PP-MC pairs, focal child = 12, bystander = 26, focal-bystander dyad = 51) in Analysis 3 (recalculation of Analysis 2, Model 1). In the factor "context", the parameters were shown in the same way as Table 2.
