# Supplementary material for: Preschool Children’s Behavioral Tendency toward Social Indirect Reciprocity
Source: PLoS One. 2013 Aug 7;8(8):e70915. doi: 10.1371/journal.pone.0070915 (PMC3737253; doi:10.1371/journal.pone.0070915)
Supplement: Table S6 — Influence of independent factors on the number of affiliative behavior from bystanders in Analysis 3 (recalculation of Analysis 2, Model 2). (PDF) [file pone.0070915.s006.pdf]

Table S6: Influence of independent factors on the number of affiliative behavior from bystanders in Analysis 3 (recalculation of Analysis 2, Model 2)

Independent term<br>Factors	Level	Coef	SE (coef)	*t*	*P* (>|*t*|)
Intercept		- 0.44	0.66	- 0.67	0.50
Context	PP	0.42	0.13	3.17	< 0.01
Familiarity between focal children and bystanders		1.48	0.99	1.49	0.14
The focal children's usual frequency of receiving affiliative behavior		0.02	0.01	2.61	0.01

We analyzed the data in 138 sessions (69 PP-MC pairs, focal child = 12, bystander = 26, focal-bystander dyad = 51) in Analysis 3 (recalculation of Analysis 2, Model 2). In the factor "context", the parameters were shown in the same way as Table 2.
